# Application of spot vision screening combined with axial length measurement for early identification of myopia risk in Chinese Young children

**DOI:** 10.3389/fped.2026.1796785

**Published:** 2026-04-01

**Authors:** Hong Liu, Jianfeng Zhong, Yingying Li, Linhui Ge, Qian Chen, Jingyu Li

**Affiliations:** Department of Ophthalmology, Affiliated Maternity and Child Healthcare Hospital of Nantong University, Nantong, Jiangsu, China

**Keywords:** axial length, early identification, myopia, young children, spot vision screening

## Abstract

**Purpose:**

To evaluate the efficacy of Spot vision screening combined with axial length measurement for early identification of myopia risk in Chinese Young children.

**Methods:**

This cross-sectional study included 210 children aged 3–8 years, classified into a Myopia Risk Group [cycloplegic spherical equivalent, cycloplegic spherical equivalent (SE) ≤ +0.75 D] and a Sufficient Hyperopia Reserve Group (SE > +0.75 D). All participants underwent comprehensive examinations including axial length, anterior chamber depth (ACD), Spot vision screening, auto-refraction, and cycloplegic refraction.

**Results:**

The Myopia Risk Group was significantly older and had a higher prevalence of parental myopia history. They also exhibited significantly longer axial length, deeper ACD, higher intraocular pressure, and higher best-corrected visual acuity. Refractive measurements from both the Spot screener and auto-refractor showed significantly lower spherical power, cylindrical power, and spherical equivalent values in the Myopia Risk Group (all *P* < 0.05). Multivariable analysis identified axial length as an independent risk factor (OR = 4.73, *P* = 0.002). Spot SE showed a strong correlation with cycloplegic SE (r = 0.85, *P* < 0.001) and demonstrated excellent predictive value for myopia risk (AUC = 0.931). The combination of axial length and Spot SE achieved an AUC of 0.956 for identifying children at risk of myopia.

**Conclusion:**

The combination of Spot vision screening and axial length measurement effectively identifies young children at risk for myopia, supporting its use in early screening programs.

## 1 Introduction

Myopia, or nearsightedness, is a common refractive error that has become a significant public health concern globally (1, 2). It affects millions of people, particularly in East Asia, where the prevalence among school-aged children has risen dramatically over recent decades (3). Early onset of myopia during childhood increases the risk of developing high myopia later in life, which can lead to serious complications such as retinal detachment, glaucoma, and macular degeneration. Therefore, accurately identifying children at risk of myopia at an early age is a critical first step toward implementing potential preventive measures (4, 5).

Early detection of myopia risk factors allows for timely interventions that may slow down or prevent the progression of myopia. Several methods have been used to screen for myopia, including visual acuity tests and cycloplegic refraction. However, these methods often require cooperation from the child and may not be suitable for very young children. Moreover, traditional screening methods might miss subtle changes in refractive status that could indicate early stages of myopia development (6–8). Recent advancements in technology have introduced new tools like the Spot vision screener, which provides quick and non-invasive assessments of refractive errors (9).

Axial length is one of the most important predictors of myopia. As the eye grows longer, light focuses in front of the retina rather than on it, resulting in myopia. Monitoring axial length changes over time can provide insights into the progression of myopia and help identify children at higher risk. Studies have shown that even small increases in axial length can be associated with significant changes in refractive error (10, 11). Thus, integrating axial length measurements with refractive data from devices like the Spot vision screener could offer a more comprehensive approach to myopia risk assessment (12). This combination may be particularly useful in young children, who are at a critical stage for detecting early signs of myopia before it becomes clinically apparent.

Young children represent a unique population for myopia screening due to their rapid eye growth and development. During this period, subtle changes in ocular parameters can signal the beginning of myopia (13, 14). Early intervention in this group can have a substantial impact on preventing or delaying the onset of myopia. However, conducting detailed eye examinations in young children presents challenges, including difficulties with cooperation and compliance. The Spot vision screener addresses some of these issues by providing rapid assessments that require minimal participation from the child. When combined with axial length measurement, this approach offers a promising method for identifying myopia risk in young children. Such screenings can facilitate early detection and timely management strategies, potentially reducing the burden of myopia in later years. This study aims to explore the effectiveness of combining Spot vision screening with axial length measurement for early identification of myopia risk in Chinese young children, contributing to the broader effort of mitigating the impact of myopia on public health.

## 2 Materials and methods

### 2.1 Study design and participants

This retrospective, cross-sectional study was conducted at the Department of Ophthalmology, Nantong Maternal and Child Health Hospital. The study protocol adhered to the tenets of the Declaration of Helsinki and was approved by the hospital's Institutional Review Board, with a waiver for informed consent due to the retrospective nature of the analysis.

We reviewed the medical records of children who underwent comprehensive ophthalmological examinations between June 2023 and July 2025. The initial screening identified 230 children. A final cohort of 210 children (210 right eyes) was included in the analysis.

Inclusion Criteria: ① Children aged 3 to 8 years. ② Availability of complete examination records, including Spot vision screening, ocular biometry, and cycloplegic refraction. Exclusion Criteria: ① Presence of organic ocular diseases (e.g., cataract, corneal opacity, retinopathy of prematurity). ② History of strabismus or amblyopia. ③ Previous ocular surgery or trauma. ④ Current use of orthokeratology lenses or other myopia control interventions.

### 2.2 Group definition

Based on the cycloplegic spherical equivalent (SE), which served as the reference standard, all participants were defined into one of two distinct groups. The Myopia Risk Group was defined by a cycloplegic SE of +0.75 diopters or less. This cohort encompassed children who were already myopic, with an SE of −0.50 D or less, as well as those considered pre-myopic, with an SE greater than −0.50 D but not exceeding +0.75 D. According to the International Myopia Institute (IMI) classification, both pre-myopia and myopia represent states of insufficient hyperopic reserve and are associated with an increased likelihood of progression to higher myopia. Merging these two categories into a single “Myopia Risk Group” is clinically meaningful because it identifies all children who may benefit from early monitoring or intervention, irrespective of whether they have already crossed the myopia threshold. The Sufficient Hyperopia Reserve Group, which served as the reference, included all children whose cycloplegic SE was greater than +0.75 D. These children were considered to possess a level of hyperopic reserve that is adequate for their age (15).

### 2.3 Data collection, examination procedures and measurements

Demographic and baseline characteristics, including age, gender, BMI (body mass index), parental myopia history, gestational age, and birth weight, were extracted from the standardized electronic medical records. All examinations were performed by trained ophthalmologists and optometrists according to standardized clinical protocols.

Uncorrected Visual Acuity (UCVA) and Best-Corrected Visual Acuity (BCVA) were assessed using a standard logarithmic visual acuity chart and recorded in decimal notation. Intraocular Pressure was measured using a non-contact tonometer.

Axial length (AL), corneal curvature (flat keratometry K1 and steep keratometry K2), and anterior chamber depth (ACD) were measured using the IOLMaster 500 (Carl Zeiss Meditec, Germany). A minimum of five measurements were taken for each eye, and the average value was used for analysis.

The Welch Allyn Spot™ vision screener was used for rapid, non-cycloplegic refractive screening. With the child in a seated position, the handheld device was operated at a distance of approximately 1 meter in a semi-darkened room. The device's built-in audiovisual targets (bird sounds and flashing lights) were used to attract the child's fixation. Measurements for spherical power (S), cylindrical power (C), axis (A), and spherical equivalent (SE) for both eyes were obtained automatically within seconds.

An auto kerato-refractometer (KR−800, Topcon Corporation, Japan) was used to obtain non-cycloplegic refractive measurements (spherical power, cylindrical power, axis) prior to cycloplegia.

Cycloplegia was induced to obtain the gold-standard refractive measurement. Regimens of 1% atropine sulfate ophthalmic gel (Shenyang Xingqi) were used based on clinical preference and the child's age. Once daily for 7 consecutive days or twice daily for 5 consecutive days. Parents were instructed to compress the nasolacrimal duct for 2–3 min after administration to minimize systemic absorption. Cycloplegia was considered complete when the pupil was dilated to >6.0 mm and the light reflex was absent. The distribution of the two atropine regimens was balanced between the Sufficient Hyperopia Reserve Group and the Myopia Risk Group, and the refractive measurements obtained under the two regimens were comparable, ensuring the consistency of cycloplegic refraction results across all participants. Retinoscopy was then performed by an experienced optometrist who was blinded to the results of the Spot screening and auto-refraction (16).

All examinations were performed by trained ophthalmologists and optometrists according to standardized clinical protocols. To ensure measurement reliability, all devices were calibrated daily according to the manufacturers' instructions. We performed repeat measurements on a subset of 20 children within the same visit, and the intraclass correlation coefficients for axial length and Spot SE were 0.998 and 0.985, respectively, confirming excellent repeatability.

### 2.4 Data cleaning

Before initiating data analysis, this study adhered to a rigorous data cleaning protocol aimed at identifying and rectifying any inconsistencies, errors, or missing values within the dataset. The comprehensive process included meticulous inspection of the dataset, removal of duplicate entries, correction of data entry inaccuracies, and systematic handling of missing values.

To address missing data, multiple methodologies were employed: ① Utilizing Python 3.6.0, the datawig and pandas libraries were employed to predict and impute missing values through deep neural networks. ② In Python 3.6.0, stochastic regression imputation was conducted using the pandas, numpy, seaborn, random, and missingno libraries. ③ Within R 4.3.2, the mice package was utilized for implementing MICE, a robust and flexible approach for handling missing data across various types of variables. ④ Employing Python 3.6.0 and the Impyute library, KNN-based imputation was performed. Initially, a basic mean imputation was established, followed by constructing a KDTree from the complete list. The nearest neighbors were identified using the KDTree, and their weighted average was used to fill in the missing values.

To maintain data integrity, imputation was only applied when the proportion of missing data did not exceed 5%, thereby minimizing the risk of selection bias. Furthermore, sensitivity analyses were conducted by assuming the outcomes of lost-to-follow-up cases as both worst-case and best-case scenarios. If no significant differences were observed between these extreme scenarios, it was concluded that attrition had minimal impact on the final results, thus validating the robustness of the conclusions drawn. The final results presented were based on the dataset after missing value imputation.

The overall proportion of missing data was low, with all variables having less than 2% missing values (overall completeness 98.5%). To ensure robustness, we performed a complete-case analysis on the 207 children with no missing data; the results were consistent with those obtained after multiple imputation, indicating that imputation did not introduce bias. Although the overall missing rate was low (<2%), multiple imputation was employed primarily as a sensitivity analysis to validate the robustness of the complete-case findings. The consistency between the results obtained from the imputed dataset and those from the complete-case analysis (*n* = 207) confirms that the handling of missing data did not bias the conclusions. This approach aligns with recommended practices for transparency and methodological rigor.

### 2.5 Statistical analysis

All statistical analyses were performed using R software (version 4.3.2; R Foundation for Statistical Computing, Vienna, Austria). Only data from the right eye of each participant were included to avoid the bias of inter-eye correlation. Continuous variables were tested for normality using the Shapiro–Wilk test. Normally distributed data were presented as mean ± standard deviation, while non-normally distributed data were presented as median [interquartile range]. Categorical variables were expressed as frequencies and percentages (%). The Wilcoxon rank sum test was used for continuous non-normally distributed variables, and the independent samples *t*-test was used for normally distributed variables. The Chi-square test was used for categorical variables.

Univariate and multivariable logistic regression analyses were performed to identify factors independently associated with myopia risk. Odds ratios (ORs) and their 95% confidence intervals (CIs) were calculated. The events-per-variable (EPV) ratio for the multivariable logistic regression model was calculated as 11.1 (89 events in the Myopia Risk Group divided by 8 predictor variables), which exceeds the commonly recommended threshold of 10, indicating adequate sample size and model stability. A two-tailed *P*-value of <0.05 was considered statistically significant. The diagnostic efficacy of Spot SE and axial length for identifying the Myopia Risk Group was evaluated using Receiver Operating Characteristic (ROC) curve analysis. The area under the curve (AUC), sensitivity, specificity, and Youden's index were calculated to determine optimal cut-off values. The ten-fold cross-validation was performed on the final multivariable model containing the pre-specified predictors (selected based on univariate analysis and clinical relevance). Within each fold, the model was refitted to the training set and evaluated on the validation set; no additional feature selection was conducted within the folds, thereby avoiding data leakage. The average cross-validated AUC is reported as the final performance metric.

## 3 Results

### 3.1 Baseline characteristics

Comparing the demographic and basic ocular characteristics between the Sufficient Hyperopia Reserve Group and the Myopia Risk Group (Table 1), the Myopia Risk Group was older [5 (4, 6) vs. 4 (3, 4) years, W = 7,172.000, *P* < 0.001], had a higher prevalence of parental myopia history (16.85% vs. 6.61%, *χ*^2^ = 5.516, *P* = 0.019), higher BCVA [0.9 (0.7, 1) vs. 0.7 (0.5, 0.8), W = 3,209.500, *P* < 0.001], and higher intraocular pressure [15 (13, 17) vs. 14 (12, 16) mmHg, W = 3,985.500, *P* = 0.001]. No significant differences were observed for BMI (*P* = 0.770), gender (*P* = 0.540), birth weight (*P* = 0.142), gestational age (*P* = 0.189), or uncorrected visual acuity (*P* = 0.620).

**Table 1:** Comparison of demographic and basic ocular characteristics between the two groups.

Parameters	Sufficient hyperopia reserve group (*n* = 121)	Myopia risk group (*n* = 89)	W/t/*χ*^2^	*P*
Age (years)	4 [3, 4]	5 [4, 6]	7,172.000	<0.001
BMI (kg/m^2^)	15.78 ± 1.25	15.83 ± 1.28	0.293	0.770
Gender (Boys/Girls) [*n* (%)]	56 (46.28%)/65 (53.72%)	45 (50.56%)/44 (49.44%)	0.376	0.540
Parental Myopia History [*n* (%)]	8 (6.61%)	15 (16.85%)	5.516	0.019
Birth Weight (kg)	3.34 ± 0.42	3.25 ± 0.41	1.473	0.142
Gestational Age at Birth (weeks)	39.16 ± 1.03	38.97 ± 1.07	1.319	0.189
Uncorrected Visual Acuity	0.5 [0.3, 0.6]	0.4 [0.3, 0.6]	5,599.000	0.620
Best Corrected Visual Acuity	0.7 [0.5, 0.8]	0.9 [0.7, 1]	3,209.500	<0.001
Intraocular Pressure (mmHg)	14 [12, 16]	15 [13, 17]	3,985.500	0.001

BMI, body mass index.

### 3.2 Ocular biometric parameters

Significant intergroup differences were observed for axial length and ACD (Table 2). The Myopia Risk Group demonstrated a substantially longer axial length [23.27 (22.79, 23.69)] compared to the Sufficient Hyperopia Reserve Group [21.67 (21.01, 22.29)] (*P* < 0.001). Similarly, the ACD was deeper in the Myopia Risk Group [3.34 (3.16, 3.49)] than in the reference group [3.01 (2.97, 3.21)] (*P* < 0.001). In contrast, corneal curvature parameters K1 (*P* = 0.089) and K2 (*P* = 0.627) did not differ significantly between the groups.

**Table 2:** Comparison of ocular biometric parameters between groups.

Parameters	Sufficient hyperopia reserve group (*n* = 121)	Myopia risk group (*n* = 89)	W	*P*
Axial Length (mm)	21.67 [21.01, 22.29]	23.27 [22.79, 23.69]	887.000	<0.001
Corneal Curvature, K1 (D)	42.19 [41.56, 43.44]	42.72 [41.62, 43.83]	4,645.000	0.089
Corneal Curvature, K2 (D)	44.7 [43.1, 45.98]	44.53 [43.44, 45.42]	5,596.500	0.627
ACD	3.01 [2.97, 3.21]	3.34 [3.16, 3.49]	2,450.000	<0.001

ACD, anterior chamber depth.

### 3.3 Spot vision screener measurements

**Figure 1:**
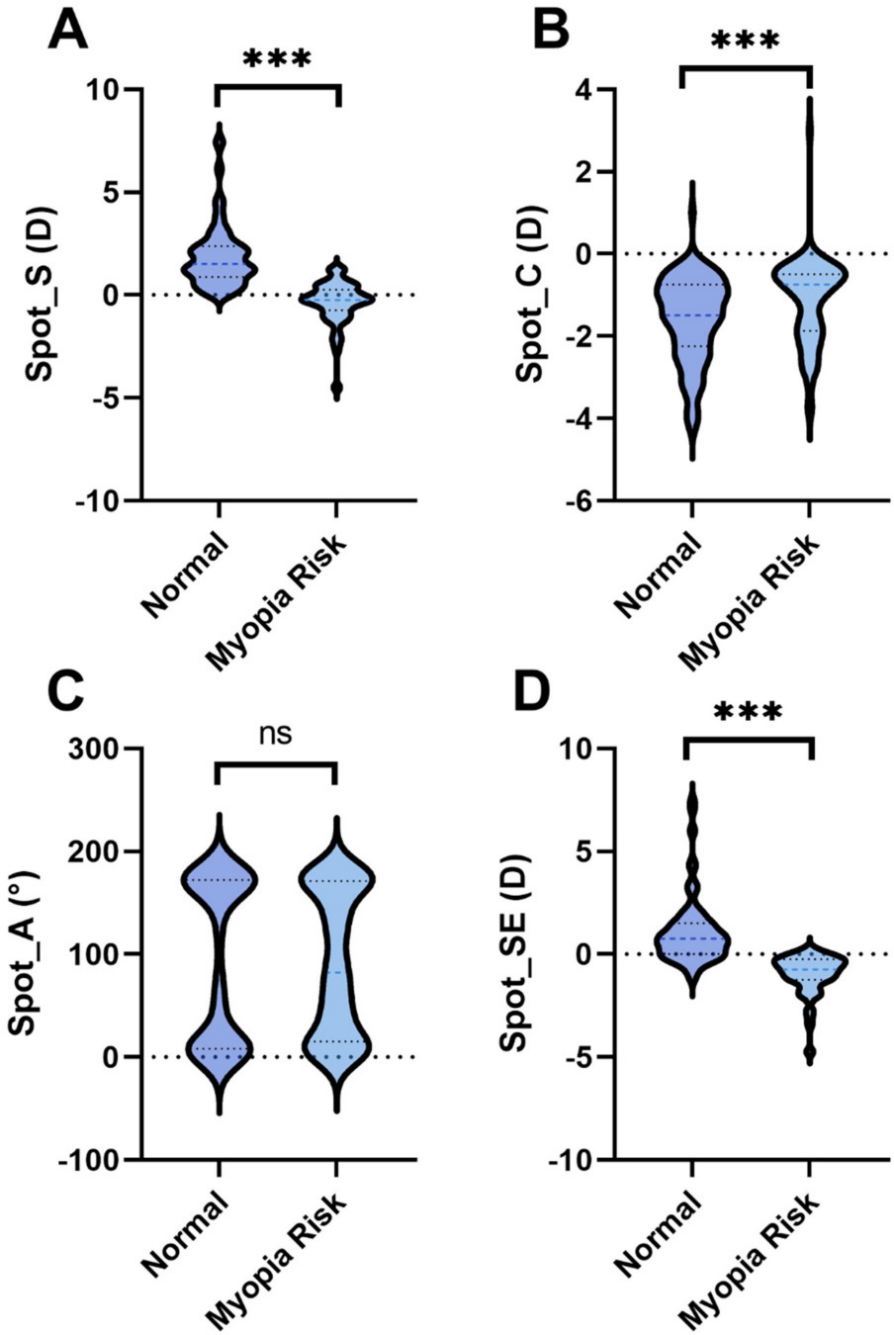
Comparison of spot vision screener measurements between groups. **(A)** Spot_S; **(B)** Spot_C; **(C)** Spot_A; **(D)** Spot_SE. ns: no significant difference; ***: *P* < 0.001. S, spherical power; C, cylindrical power; A, axis; SE, spherical equivalent.

The Spot vision screener parameters S, C, and SE all showed highly significant differences (all *P* < 0.001) (Figure 1). The Myopia Risk Group had a lower median S [−0.25 (−0.75, 0.25)] and SE [−0.75 (−1.25, −0.25)] compared to the Sufficient Hyperopia Reserve Group [S: 1.5 (1, 2.25); SE: 0.75 (0, 1.5)]. The median C was less negative in the Myopia Risk Group [−0.75 (−1.75, −0.5)] than in the reference group [−1.5 (−2.25, −0.75)]. The parameter A did not differ between groups (*P* = 0.987).

### 3.4 Auto-refractor measurements

Auto-refractor measurements revealed that S and SE were significantly lower in the Myopia Risk Group [S: −1 (−1.5, −0.25); SE: −1.25 (−2.25, −1)] than in the Sufficient Hyperopia Reserve Group [S: 1.25 (0.5, 2.5); SE: 0.5 (−0.25, 1.75)] (both *P* < 0.001) (Table 3). A significant difference was also found for C (*P* = 0.021), which was less negative in the Myopia Risk Group [−0.75 (−1.75, −0.5)] than in the reference group [−1.25 (−2, −0.5)]. The parameter A showed no significant difference (*P* = 0.465).

**Table 3:** Comparison of auto-refractor measurements between groups.

Parameters	Sufficient hyperopia reserve group (*n* = 121)	Myopia risk group (*n* = 89)	W	*P*
Auto_Refractor_S (D)	1.25 [0.5, 2.5]	−1 [−1.5, −0.25]	9,992.500	<0.001
Auto_Refractor_C (D)	−1.25 [−2, −0.5]	−0.75 [−1.75, −0.5]	4,384.000	0.021
Auto_Refractor_A (°)	115 [10, 173]	124 [15, 175]	5,066.500	0.465
Auto_Refractor_SE (D)	0.5 [−0.25, 1.75]	−1.25 [−2.25, −1]	9,813.000	<0.001

S, spherical power; C, cylindrical power; A, axis; SE, spherical equivalent.

### 3.5 Cycloplegic refraction measurements

Consistent with other refractive measures, cycloplegic S and SE were markedly lower in the Myopia Risk Group [S: 0 (−0.5, 0.75); SE: −0.5 (−1, 0)] compared to the Sufficient Hyperopia Reserve Group [S: 3.25 (2.25, 4.75); SE: 2.5 (1.75, 4.25)] (both *P* < 0.001) (Table 4). A significant intergroup difference was also noted for C (*P* = 0.037), while the parameter A was not significantly different (*P* = 0.446).

**Table 4:** Comparison of cycloplegic refraction measurements between groups.

Parameters	Sufficient hyperopia reserve group (*n* = 121)	Myopia risk group (*n* = 89)	W	*P*
Cycloplegic_S (D)	3.25 [2.25, 4.75]	0 [−0.5, 0.75]	10,677.000	<0.001
Cycloplegic_C (D)	−1.25 [−2, −0.5]	−0.75 [−1.5, −0.5]	4,480.000	0.037
Cycloplegic_A (°)	164 [10, 178]	140 [12, 175]	5,713.500	0.446
Cycloplegic_SE (D)	2.5 [1.75, 4.25]	−0.5 [−1, 0]	10,769.000	<0.001

S, spherical power; C, cylindrical power; A, axis; SE, spherical equivalent.

### 3.6 Univariate and multivariable logistic regression analysis

In the univariate analysis, several factors demonstrated significant associations with myopia risk (Table 5). Higher values of BCVA (OR = 27.41), intraocular pressure (OR = 1.24), AL (OR = 10.27), ACD (OR = 25.46), and Spot_C (OR = 1.57) were associated with increased risk of myopia. Conversely, higher values of Spot_S (OR = 0.06), Spot_SE (OR = 0.04), and Auto_Refractor_S (OR = 0.20) were associated with decreased risk of myopia. The remaining factors, including age, parental myopia history, Auto_Refractor_C, Auto_Refractor_SE, Cycloplegic_S, and Cycloplegic_C, showed no statistically significant associations (all *P* > 0.05).

**Table 5:** Univariate analysis of factors associated with myopia risk.

Parameters	Coefficient	Std_Error	Wald_Stat	OR	OR_95% CI	*P*
Age	0.655	0.381	1.719	1.925	0.804–2.246	0.085
Parental Myopia History	1.052	0.463	2.274	2.863	1.183- 7.427	0.053
Best Corrected Visual Acuity	3.311	0.727	4.554	27.411	6.957–121.394	<0.001
Intraocular Pressure	0.212	0.062	3.386	1.236	1.097–1.402	0.001
Axial Length	2.330	0.309	7.549	10.274	5.891–19.870	<0.001
ACD	3.237	0.649	4.991	25.459	7.676–97.793	<0.001
Spot_S	−2.790	0.408	−6.839	0.061	0.025–0.126	<0.001
Spot_C	0.448	0.149	3.007	1.565	1.180–2.120	0.003
Spot_SE	−3.153	0.481	−6.554	0.043	0.015–0.099	<0.001
Auto_Refractor_S	−1.608	0.236	−6.808	0.200	0.121–0.306	<0.001
Auto_Refractor_C	0.225	0.157	1.428	1.252	0.924–1.717	0.153
Auto_Refractor_SE	−1.583	0.239	−6.624	0.205	0.123–0.315	0.174
Cycloplegic_S	−0.163	0.149	−1.091	0.845	0.634–1.164	0.276
Cycloplegic_C	0.204	0.159	1.285	1.226	0.902–1.685	0.199

ACD, anterior chamber depth; S, spherical power; C, cylindrical power; A, axis; SE, spherical equivalent.

Variables demonstrating significant associations in the univariate analysis were included in the multivariable logistic regression model (Table 6). Due to the linear correlation between Spot_S and Spot_SE, only Spot_SE was incorporated into the multivariable analysis. The results identified axial length as an independent risk factor for myopia (OR = 4.73, 95% CI: 1.77–12.66, *P* = 0.002), while Spot_SE were associated with decreased risk of myopia (OR = 0.05, 95% CI: 0.01–0.22, *P* < 0.001). The other variables included in the model as Age, Best Corrected Visual Acuity, intraocular pressure, ACD, Spot_C, and Auto_Refractor_S showed no significant independent associations with myopia risk (all *P* > 0.05).

**Table 6:** Multivariable logistic regression analysis of factors associated with myopia risk.

Parameters	Coefficient	Std_Error	Wald_Stat	OR	OR_CI_Lower	OR_CI_Upper	*P*
Age	0.095	0.281	0.338	1.100	0.634	1.909	0.735
Best Corrected Visual Acuity	2.050	2.215	0.926	7.768	0.101	596.583	0.355
Intraocular Pressure	0.204	0.162	1.255	1.226	0.892	1.684	0.209
Axial Length	1.554	0.502	3.096	4.731	1.769	12.655	0.002
ACD	2.308	1.250	1.847	10.051	0.868	116.389	0.065
Spot_C	−0.134	0.420	−0.320	0.874	0.384	1.990	0.749
Spot_SE	−3.007	0.767	−3.920	0.049	0.011	0.222	<0.001
Auto_Refractor_S	−1.222	0.406	−3.012	0.295	0.133	0.653	0.053

ACD, anterior chamber depth; S, spherical power; C, cylindrical power; A, axis; SE, spherical equivalent.

Pearson correlation analysis showed that axial length was strongly negatively correlated with cycloplegic spherical equivalent (r = −0.78, *P* < 0.001), while Spot SE was strongly positively correlated with cycloplegic SE (r = 0.85, *P* < 0.001). To assess multicollinearity in the multivariable model, we calculated variance inflation factors (VIFs) for all predictors; all VIFs were below 5, indicating no serious collinearity.

### 3.7 ROC analysis

**Figure 2:**
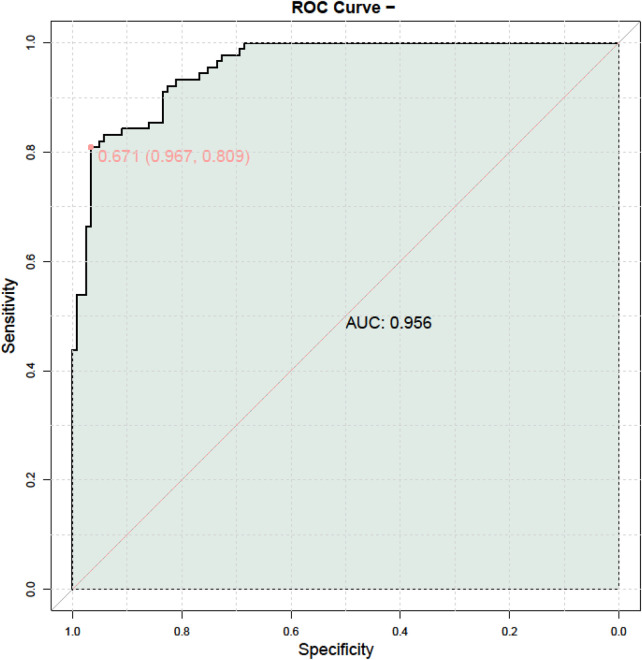
Joint ROC curve. ROC, receiver operating characteristic.

ROC analysis identified axial length (AUC = 0.918) and Spot_SE (AUC = 0.931) as the two most powerful discriminators for myopia risk (Table 7). Spot_SE achieved a high sensitivity of 0.97 at its optimal cut-off of 0.125. Auto_Refractor_S (AUC = 0.828) and ACD (AUC = 0.772) also demonstrated good diagnostic performance. Other parameters, such as BCVA (AUC = 0.702), intraocular pressure (AUC = 0.630), and Spot_C (AUC = 0.636), showed moderate to low discriminatory ability. Based on the two independent predictors identified in the multivariable analysis (axial length and Spot_SE), a joint prediction model was constructed. The joint ROC curve presented in Figure 2 visually demonstrated the enhanced diagnostic efficacy of this combined model, which achieved an AUC of 0.956 (95% CI: 0.924–0.988), surpassing the AUC values of either individual parameter alone. This result was confirmed by ten-fold cross-validation.

**Table 7:** ROC analysis of factors associated with myopia risk.

Parameters	Best_threshold	Sensitivities	Specificities	AUC	AUC 95% CI	Youden_index
Best Corrected Visual Acuity	0.750	0.730	0.595	0.702	0.630–0.774	0.325
Intraocular Pressure	14.500	0.640	0.537	0.630	0.555–0.705	0.177
Axial Length	22.690	0.809	0.851	0.918	0.878–0.958	0.660
ACD	3.305	0.607	0.901	0.772	0.708–0.836	0.508
Spot_C	−0.875	0.528	0.727	0.636	0.560–0.712	0.255
Spot_SE	0.125	0.966	0.727	0.931	0.892–0.970	0.693
Auto_Refractor_S	0.125	0.910	0.868	0.828	0.774–0.882	0.778

ACD, anterior chamber depth; S, spherical power; C, cylindrical power; A, axis; SE, spherical equivalent.

## 4 Discussion

Early identification of myopia risk in young children is widely regarded as a crucial step for implementing potential preventive measures and may ultimately help reduce the long-term burden of myopia. This study explored the application of Spot vision screening combined with axial length measurement to detect myopia risk in Chinese young children. Our findings indicate that several ocular biometric parameters, including axial length and ACD, along with refractive measurements from Spot vision screener and auto-refractor, are associated with myopia.

Children at risk of myopia were older and had a higher prevalence of parental myopia history compared to those with sufficient hyperopia reserve. Age is a well-known risk factor for myopia development, as the eye grows during childhood and adolescence, leading to elongation of the axial length. Parental myopia history also plays a role, as genetic factors contribute to the likelihood of developing myopia. These demographic characteristics highlight the importance of early screening in younger children, especially those with a family history of myopia (17, 18). The higher intraocular pressure observed in the Myopia Risk Group may be an early indicator of ocular changes associated with myopia progression. In contrast, the higher BCVA in this group is likely attributable to the age difference, as younger children (3-4 years) typically have lower visual acuity due to normal developmental processes, while older children (5-6 years) achieve higher acuity. Thus, BCVA was not considered a direct indicator of myopia risk in this study.

Axial length was longer in the Myopia Risk Group, indicating that increased axial length is a key factor in the development of myopia. Axial length elongation is one of the primary mechanisms leading to myopia, as it causes light to focus in front of the retina rather than on it, resulting in blurred distance vision (19–21). A deeper ACD was also observed in the Myopia Risk Group. The relationship between ACD and myopia is complex; however, a deeper ACD can be associated with a more prolate shape of the eye, which contributes to axial elongation (22–24). These findings align with previous studies showing that axial length is a strong predictor of myopia onset and progression. For example, a study by Liu et al. (25) demonstrated that axial length growth accelerates significantly after myopia onset, particularly in children younger than 10.

The Spot vision screener measurements showed differences in SE, S, and C between the two groups. Lower SE and S values in the Myopia Risk Group suggest a trend towards myopia, characterized by a shift from hyperopia to emmetropia or myopia. This shift indicates that the eye is becoming longer or the cornea steeper, leading to light focusing in front of the retina rather than directly on it. The cylinder values were less negative in the Myopia Risk Group, indicating a possible change in corneal curvature or lens power contributing to myopia development (26, 27). Changes in corneal curvature can result from alterations in collagen fiber arrangement or thickness, which may be influenced by genetic factors or environmental conditions such as prolonged near work. Additionally, variations in lens power could be due to changes in the lens's refractive index or its positioning within the eye, potentially driven by accommodative stress or other biomechanical factors. The Spot vision screener provides a quick and non-invasive method for assessing refractive errors, making it suitable for large-scale screenings in young children (28). Its ability to detect subtle changes in refractive status highlights its potential utility in identifying children at risk of myopia, allowing for early intervention strategies to mitigate the progression of myopia (29). By capturing these early signs, clinicians can better understand the underlying mechanisms driving myopia development and tailor treatments accordingly.

Similar trends were observed in auto-refractor measurements, with lower SE and S values in the Myopia Risk Group. These findings reinforce the importance of accurate refractive measurements in early myopia detection. The auto-refractor provides more detailed information about the refractive status of the eye, allowing for a more comprehensive assessment of myopia risk. However, it requires cooperation from the child, which can be challenging in younger populations (14, 30). Combining Spot vision screening with auto-refractor measurements could enhance the accuracy of myopia risk assessments by providing complementary data.

Univariate analysis identified several factors associated with myopia risk, including BCVA, intraocular pressure, axial length, ACD, and Spot_C. These factors reflect various aspects of ocular health and structure that contribute to myopia development. Multivariable logistic regression revealed axial length as an independent risk factor for myopia. Axial length is a robust predictor of myopia due to its direct impact on the optical properties of the eye. Spot_SE showed a strong positive correlation with cycloplegic SE, which reflects the spherical equivalent, indicates the overall refractive status and helps identify children who may benefit from early interventions. The combination of these two parameters in a joint prediction model enhanced diagnostic efficacy, suggesting that integrating multiple measurements can improve the accuracy of myopia risk assessment.

Despite providing valuable insights, this study has several limitations. It was conducted in a single center, which may limit the generalizability of the findings. Due to the cross-sectional design, causal relationships cannot be established, and our findings are limited to identifying current myopia risk rather than predicting future onset. Longitudinal studies are needed to confirm the predictive value of these parameters. Future studies should include a larger and more diverse population to validate these results. The study focused on young children, but it would be beneficial to investigate whether these findings apply to older age groups or different ethnicities. Although age differed significantly between groups and is known to influence ocular development, we did not perform stratified analyses by age subgroups due to sample size limitations. Future studies with larger cohorts are warranted to explore whether the predictive performance of axial length and Spot SE varies across different age strata within the young population. Lastly, while our study examined several ocular parameters, additional factors such as environmental influences and lifestyle habits could also contribute to myopia development. Further research is needed to explore these factors and develop comprehensive strategies for myopia prevention.

This study demonstrates the potential of Spot vision screening combined with axial length measurement for early identification of myopia risk in Chinese young children. Axial length and Spot_SE emerged as key predictors of myopia, highlighting their importance in clinical practice. Integrating multiple ocular biometric and refractive parameters can enhance the accuracy of myopia risk assessments, enabling timely interventions to prevent or delay myopia onset. Continued research in this area will provide valuable insights into the mechanisms underlying myopia development and help develop effective strategies for managing this growing public health concern.

## 5 Conclusion

This cross-sectional study suggests that the combination of Spot vision screening and axial length measurement may be effective for early identification of myopia risk in Chinese young children. The findings indicate that children with longer axial length and lower Spot SE are more likely to be at risk, highlighting the potential of these parameters in screening programs. These parameters, particularly axial length, appear to be key indicators of myopia development, reflecting underlying changes in ocular structure and refractive status. Additionally, the Spot vision screener's ability to detect subtle changes in refractive error highlights its utility in large-scale screenings among young children. Integrating multiple ocular biometric and refractive parameters may enhance the accuracy of myopia risk assessments, providing valuable insights for timely interventions.

## Data Availability

The raw data supporting the conclusions of this article will be made available by the authors, without undue reservation.
